# Relation of severe COVID-19 in Scotland to transmission-related factors and risk conditions eligible for shielding support: REACT-SCOT case-control study

**DOI:** 10.1186/s12916-021-02021-5

**Published:** 2021-06-23

**Authors:** Paul M. McKeigue, David A. McAllister, David Caldwell, Ciara Gribben, Jen Bishop, Stuart McGurnaghan, Matthew Armstrong, Joke Delvaux, Sam Colville, Sharon Hutchinson, Chris Robertson, Nazir Lone, Jim McMenamin, David Goldberg, Helen M. Colhoun

**Affiliations:** 1 Usher Institute, College of Medicine and Veterinary Medicine, University of Edinburgh, Teviot Place, Edinburgh, EH8 9AG Scotland; 2 Public Health Scotland, Meridian Court, 5 Cadogan Street, Glasgow, G2 6QE Scotland; 3 Institute of Health and Wellbeing, University of Glasgow, 1 Lilybank Gardens, Glasgow, G12 8RZ Scotland; 4 School of Health and Life Sciences, Glasgow Caledonian University, Glasgow, Scotland; 5 Department of Mathematics and Statistics, University of Strathclyde, 16 Richmond Street, Glasgow, G1 1XQ Scotland; 6 Institute of Genetics and Molecular Medicine, College of Medicine and Veterinary Medicine, University of Edinburgh, Western General Hospital Campus, Crewe Road, Edinburgh, EH4 2XUC Scotland

**Keywords:** COVID-19/prevention and control, Transplant recipient, Nosocomial infection, Matched case control Studies

## Abstract

**Background:**

Clinically vulnerable individuals have been advised to shield themselves during the COVID-19 epidemic. The objectives of this study were to investigate (1) the rate ratio of severe COVID-19 associated with eligibility for the shielding programme in Scotland across the first and second waves of the epidemic and (2) the relation of severe COVID-19 to transmission-related factors in those in shielding and the general population.

**Methods:**

In a matched case-control design, all 178,578 diagnosed cases of COVID-19 in Scotland from 1 March 2020 to 18 February 2021 were matched for age, sex and primary care practice to 1,744,283 controls from the general population. This dataset (REACT-SCOT) was linked to the list of 212,702 individuals identified as eligible for shielding. Severe COVID-19 was defined as cases that entered critical care or were fatal. Rate ratios were estimated by conditional logistic regression.

**Results:**

With those without risk conditions as reference category, the univariate rate ratio for severe COVID-19 was 3.21 (95% CI 3.01 to 3.41) in those with moderate risk conditions and 6.3 (95% CI 5.8 to 6.8) in those eligible for shielding. The highest rate was in solid organ transplant recipients: rate ratio 13.4 (95% CI 9.6 to 18.8). Risk of severe COVID-19 increased with the number of adults but decreased with the number of school-age children in the household. Severe COVID-19 was strongly associated with recent exposure to hospital (defined as 5 to 14 days before presentation date): rate ratio 12.3 (95% CI 11.5 to 13.2) overall. The population attributable risk fraction for recent exposure to hospital peaked at 50% in May 2020 and again at 65% in December 2020.

**Conclusions:**

The effectiveness of shielding vulnerable individuals was limited by the inability to control transmission in hospital and from other adults in the household. Mitigating the impact of the epidemic requires control of nosocomial transmission.

**Supplementary Information:**

The online version contains supplementary material available at (10.1186/s12916-021-02021-5).

## Background

The SARS-CoV-2 pandemic reached Scotland in early 2020 with the first positive laboratory test recorded on 1 March 2020. Population-wide interventions included advice that symptomatic individuals should self-isolate, banning of mass gatherings, closure of schools and finally a population-wide lockdown on 23 March 2020. Although no systematic studies of risk factors were available at that time, public health agencies issued lists of “moderate risk” conditions [1] and “diseases and conditions considered to be very high risk” [2]. Those with “very high risk” conditions were designated as eligible for shielding and were sent letters advising strict isolation, even from other members of the same household, and offering support which included a national opt-in scheme of free food delivery, home delivery of medication and priority access to supermarket delivery slots. Those living in multiple-occupancy households were advised to isolate from other household members. Compliance was voluntary.

For this study, the research question was to investigate the effectiveness of the shielding programme individuals and to identify avoidable factors that might have limited the effectiveness of this focused protection of vulnerable individuals. The original aim was to investigate whether shielding advice and support had reduced the risk of COVID-19. The first objective was to quantify the incidence of severe COVID-19 in those eligible for shielding and to determine whether the rate ratio associated with eligibility for shielding compared to those without risk conditions had fallen after the receipt of shielding letters. The second objective was to understand the relation of severe COVID-19 in those eligible for shielding to transmission-related factors including household composition and recent exposure to hospital. As the relevance of these transmission-related factors among those eligible for shielding became clear, the aims were broadened to investigate the relation of severe COVID-19 to these transmission-related factors in the general population.

## Methods

### Overview

The original protocol for the REACT-SCOT case-control study was registered with the European Network of Centres for Pharmacoepidemiology and Pharmacovigilance (ENCEPP number EUPAS35558). We linked a national dataset of those eligible for shielding to a case-control dataset (REACT-SCOT) established in Public Health Scotland (PHS) that includes all persons diagnosed with COVID-19 in Scotland. Each case was matched to up to 10 controls of the same sex and in the same 1-year age band, registered with the same primary care practice and not previously diagnosed with COVID-19, who were alive on the date of presentation of the case controls [3]. This case-control dataset is refreshed every few weeks and linked to health records that are used to assign a list of designated “moderate risk conditions”. Thus all cases and controls were classified into three categories: no risk condition, moderate risk condition only and eligible for shielding. As a first step, we report the cumulative incidence of diagnosed COVID-19 among those in shielding. For all tests of association and comparisons of rates between groups, we limit the analysis to severe cases and their respective controls as described below, and we exclude care home residents.

The study period was from 1 March 2020 (the date of the first case in Scotland) to 18 February 2021 (date of the latest data extract available for this study). Thus, it predates the start of the shielding programme.

### Ascertainment of eligibility for shielding

The list of those eligible for shielding was generated by Public Health Scotland from March 2020 onwards by querying health-care information systems including hospital discharge records, prescription encashments, regional cancer chemotherapy databases, blood and transplant registries, for a designated list of diseases and conditions, supplemented direct requests to clinicians in relevant specialties [4]. The categories designated as eligible for shielding were as listed below [2, 4]:

1. Solid organ transplant recipient
2. Cancer of the blood or bone marrow at any stage of treatment, or people with cancer receiving treatments that affect the immune system
3. Severe respiratory conditions including cystic fibrosis, severe asthma and severe chronic obstructive airway disease, on home oxygen, severe bronchiectasis, pulmonary hypertension)
4. Rare diseases that increase the risk of infections such as severe combined immunodeficiency and homozygous sickle-cell disease
5. People on immunosuppression therapies sufficient to increase risk of infection
6. Pregnant with heart disease
7. Additional conditions, including people on renal dialysis, those who had a splenectomy and others identified by clinicians as requiring shielding advice.

The first batch of shielding letters was sent on 3 April 2020. Further batches were issued on a weekly basis and the programme was paused on 1 August 2020. On 25 November 2020, a further letter was issued with “extra protection level advice for people at highest risk” based on the current protection level for the population level in that area. The list of those eligible for shielding has been regularly updated: this study is based on the list of 212,702 individuals identified up to 28 January 2021.

### Ascertainment of cases and sampling of controls

Case ascertainment for the REACT-SCOT study has been described in detail elsewhere [3]. Case ascertainment was based on querying the following national-level databases: Electronic Communication of Surveillance in Scotland (ECOSS) that captures virology testing in all NHS laboratories, National Records of Scotland (NRS) death registrations, RAPID which is a daily update of hospitalisations and Scottish Morbidity Record 01 (SMR01) which records general hospital discharges including day cases and is ICD-10 coded. All these databases use the Community Health Index (CHI) number as identifier. The CHI database includes age, sex, postcode and care home status and can be queried to extract numbers of adults and children in the household.

Cases diagnosed with COVID-19 were defined as those with a positive nucleic acid test for SARS-CoV-2 in ECOSS, a hospital discharge diagnosis of COVID-19 in SMR01, or a death registration with mention of COVID-19 anywhere on the death certificate. Thus, not all those cases defined as having evidence of COVID-19 had a positive test. To restrict cases to those with a positive test would have missed a large proportion of deaths in the first wave as the availability of tests was restricted. Some cases with a definite clinical diagnosis of COVID-19 based on typical presentation and radiological signs nonetheless tested negative when they presented late in their illness. In the first wave (up end of August 2020), only 3136 (68%) of the severe cases were test-positive; in the second wave beginning on 1 September 2020, 5847 (96%) were test-positive.

The presentation date was assigned as the date of the first positive test for those ascertained through testing, as 7 days before the admission date for those without a positive test result ascertained through hospital discharge records, and as 14 days before the date of death for those without a positive test result ascertained through death certificates. Databases were queried from 1 March 2020 (date of the first diagnosed case of COVID-19 in Scotland) up to 18 February 2021 for test results and 12 February 2021 for deaths. For each case diagnosed with COVID-19, up to 10 community controls matched for sex, 1-year age band and primary care practice were selected from the CHI database and assigned the same presentation date as the case. With this incidence density sampling design, it is possible and correct for an individual to appear more than once as a control and subsequently as a case. As primary care practice catchment areas are localised, matching on primary care practice matches for geographic factors also.

### Inclusion and exclusion criteria for analyses

We reported the cumulative incidence of diagnosed COVID-19 in the shielded and further categorised these cases into those that required critical care or were fatal or not. We defined severe COVID-19 as those diagnosed with COVID-19 who also required entry to critical care within 21 days of presentation date, or had a fatal outcome. Entry to critical care units—intensive care, high dependency or combined units—was obtained by linkage to the Scottish Intensive Care Society and Audit Group (SICSAG) database. Fatal outcome was defined as death at any time with COVID-19 coded as underlying cause, death from any cause within 28 days of testing positive or death within 28 days of presentation date for cases ascertained only through discharge records.

For all subsequent tests of association and comparisons of rates between groups presented here, we restricted the dataset to cases with severe COVID-19 and their matched controls from the population. The narrow definition of severe COVID-19 was defined as the main outcome measure of the REACT-SCOT study at the design stage, to ensure that ascertainment would not be biased by variation in testing policies or selection for hospitalisation. In the first wave in Scotland, people with symptoms were advised to stay at home and not to seek testing or attend hospital unless their condition deteriorated. A broader case definition based on test-positive or hospitalised cases would have given rise to selection bias if, for equivalent severity of symptoms, those with pre-existing risk conditions were more likely to be tested or hospitalised than those without pre-existing risk conditions. For all tests of association and comparisons of rates between groups other than the initial tabulation of cumulative incidence of any diagnosis of COVID-19 in the shielded, we excluded care home residents because policies for shielding care home residents are different from those relevant to shielding individuals living independently.

### Linkage of cases and controls to demographic and morbidity data

Linkage of cases and controls to demographic and morbidity data and the associations of these factors with severe COVID-19 have been described in detail elsewhere [3]. Cases and controls were linked to hospital discharge ICD-10 codes over the last 5 years in SMR01, to British National Formulary codes of dispensed prescriptions in the 240 days before presentation date in the Prescribing Information System and to the national register of diabetes. We used these linked datasets to assign a list of “moderate risk conditions” for COVID-19 designated by public health agencies [1]: diabetes, heart disease, asthma or chronic airway disease, chronic kidney disease, disabling neurological conditions and immune deficiency or suppression. The codes used are as described previously [3]. Three broad risk groups were defined: no risk condition, moderate risk condition but ineligible for shielding, and eligible for shielding. Socioeconomic status was assigned as the Scottish Index of Multiple Deprivation (SIMD) score which is based on linkage of postcodes to Census data [5]; quintile 5 is the least deprived.

### Transmission-related risk factors

As addresses in the CHI database have been mapped to Unique Property Reference Numbers, it was possible to calculate the numbers of adults and children in each household and to augment the coding of care home residence in the CHI database. Care home residence was assigned using the field in the CHI database, augmented by coding as care home residents the 4548 individuals aged over 70 in households with 10 or more adults to give a total of 26,057 care home residents out of the 1,922,861 cases and controls. Linkage to occupational status for health-care workers and teachers was undertaken as described elsewhere [6, 7].

We used the Scottish Morbidity Records SMR01 (inpatients and day cases) and SMR00 (outpatient attendance) together with the RAPID database to derive variables encoding recent exposure to hospitals. We defined the variable “recent hospital exposure” as any hospital in-patient stay, day case attendance or face-to-face out-patient consultation from 5 to 14 days before presentation date. Restriction of hospital exposure to this time window was intended to exclude consultations caused by COVID-19 symptoms for which testing was delayed by a few days, but to include those exposed to health care facilities in the time interval during which the infection was likely to have been acquired. In Scotland, people with COVID-19 symptoms were instructed not to visit hospitals, to attend designated test centres for testing within 3 days and to call an emergency number for admission if their symptoms worsened. It is therefore unlikely that hospital visits by people with COVID-19 symptoms could account for the association of first positive tests 5–6 days before. As the average incubation period for COVID-19 is 5–6 days [8], exposure in the time window is relevant.

Our intention was to capture all relevant exposure in cases and controls, rather than to assign individual cases as “health-care associated COVID-19” as specified by the European Centre for Disease Prevention and Control (ECDC) [9]. The ECDC definition of probable/definite health-care-associated COVID-19 is restricted to those who have been in hospital for at least 8 days before first developing symptoms: all other cases are classified as community onset. The definition of “recent hospital exposure” used in this study was intended to capture all those whose infection could have been acquired in hospital (sensitivity of 1), unlike the ECDC definition of “probable/definite health-care associated infection” which is intended to identify those whose infection was unlikely to have been acquired outside hospital (high specificity). Because this study includes a control group, the calculation of the population attributable risk fraction is valid if the classification of exposure has sensitivity of 1 even if the specificity is less than 1 [10]. For estimating the population attributable risk fraction, it is therefore appropriate to use an inclusive definition of exposure, even if this includes some individuals whose infection was not acquired in hospital.

### Statistical methods

Other than the cumulative incidence analyses, the analyses presented here focus on severe COVID-19 and their respective controls. For the matched case-control analyses, severe cases and their respective controls were censored at date of first vaccination.

Among all severe cases and controls, conditional odds ratios for severe COVID-19 associated with eligibility for shielding and for other moderate risk conditions were calculated with “no risk condition” as reference category using conditional logistic regression. With this incidence density design, the conditional odds ratios are equivalent to rate ratios so are referred to as such throughout the manuscript. As the community controls were drawn by incidence density sampling and matched for age, sex and general practice, the conditional logistic regression inherently controls for these variables and for calendar time. Conditional logistic regression was also used to test for association of transmission-related factors with severe COVID-19. These transmission-related factors were chosen a priori as factors that would plausibly constrain the extent to which individuals were able to limit their exposure to infection: the need for hospital care, deprivation score since it may relate to overcrowding or the economic imperative to work, number of persons in the household and usual occupation. A multivariable model was used to adjust for shielding condition, moderate risk condition and transmission-related factors simultaneously. These analyses were repeated restricted to those eligible for shielding. The multivariable analyses in Tables 2 and 3 are simultaneously adjusted for all variables shown in the table.

To plot the time course of the rate ratios for severe COVID-19 associated with eligibility for shielding and with hospital exposure, these rate ratios were estimated over 21-day sliding windows of calendar time. The population attributable risk fraction of severe cases for hospital exposure was calculated in each 21-day time window from Miettinen’s formula as *p*_*c*_(*r*−1)/*r*, where *p*_*c*_ is the frequency of exposure in cases and *r* is the rate ratio [10].

Sliding windows of size 3, 7 and 21 days were used to plot the number of severe cases, the frequency of recent hospital exposure and the rate ratios associated with hospital exposure and household composition. In the plots of exposure frequency and rate ratio, the data points from 1 June 2020 to 30 September 2020 were omitted as the numbers of severe cases and controls were too low for estimates of frequencies and rate ratios to be accurate.

To test for causality of the association of recent exposure to hospital with severe COVID-19, we estimated the conditional odds ratios associated with recent hospital exposure (5 to 14 days before first testing positive) with less recent exposure (15 to 24 days before first testing positive) as reference category. This analysis was restricted to test-positive severe cases and their matched controls, so that it does not rely on the imputed presentation dates that were assigned to test-negative cases. The classic case-crossover design compares in cases only the frequencies of exposure in recent and less recent time windows and estimates the conditional odds ratio as the ratio of number of cases with recent exposure only to number of cases with less recent exposure only. This can be viewed as a matched-pairs case-control study in which the case and control are the same person in recent and less recent time windows [11]. The analysis that we report is a refinement that takes advantage of the availability of a matched control group to estimate the rate ratio for severe COVID-19 associated with exposure only in a recent time window, with those exposed only in a less recent time window as the reference category. This controls for any difference in the population frequencies of exposure or disease incidence between recent and less recent time windows.

## Results

### Shielding eligibility and risk of COVID-19

**Fig. 1:**
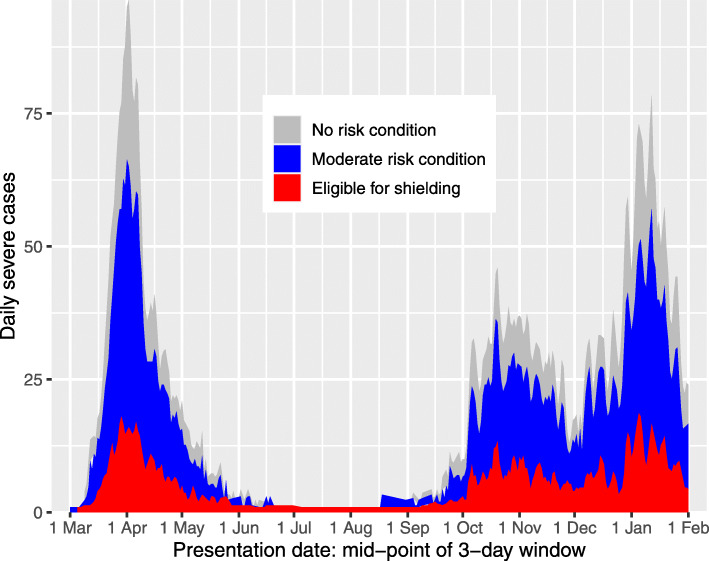
Area plot of severe cases by vulnerability category and date of presentation, excluding care home residents

Additional file 1: Table S1 shows the frequency of risk categories in those eligible for shielding. To comply with statistical disclosure control rules, the 58 women in the category “pregnant with heart disease” have been allocated to the “Additional conditions” category; there were no severe cases in this group. Table 1 shows the number in each category of eligibility for shielding by case status classified as not diagnosed, not severe or severe COVID-19; among 212,702 persons eligible for shielding, 6081 (2.9%) were diagnosed with COVID-19 but did not enter critical care or die within 28 days, 359 (0.2%) entered critical care for COVID-19 but survived and 1559 (0.7%) had a fatal outcome. As shown in Fig. 1, the time course of frequency of daily severe cases in those eligible for shielding broadly paralleled the time course in those without risk conditions. Of the 1926 severe cases among those eligible for shielding, 286 were resident in care homes.

**Table 1:** Frequencies of any diagnosis of COVID-19, entry to critical care and fatal outcome among those eligible for shielding, by eligibility category, up to 18 February 2021

		Diagnosed with COVID-19			
	Not diagnosed with COVID-19	No critical care, non-fatal	Critical care, non-fatal	Fatal	All
All shielding categories	204,703 (96.2%)	6081 (2.9%)	359 (0.2%)	1559 (0.7%)	212,644
**Shielding eligibility category**					
Solid organ transplant	6620 (96.3%)	188 (2.7%)	24 (0.3%)	40 (0.6%)	6872
Specific cancers	25,788 (96.6%)	652 (2.4%)	36 (0.1%)	223 (0.8%)	26,699
Severe respiratory	83,268 (96.2%)	2432 (2.8%)	131 (0.2%)	768 (0.9%)	86,599
Rare diseases	10,673 (96.4%)	300 (2.7%)	14 (0.1%)	85 (0.8%)	11,072
On immunosuppressants	30,796 (96.8%)	866 (2.7%)	42 (0.1%)	103 (0.3%)	31,807
Additional conditions	47,501 (95.8%)	1642 (3.3%)	112 (0.2%)	340 (0.7%)	49,595

Percentages are row percentages

Severe cases are those that entered critical care or were fatal

### Rate ratios for severe COVID-19 by risk group

Table 2 shows the rate ratios for severe COVID-19 associated with each category of shielding eligibility, with those without risk conditions as reference category, excluding care home residents. The univariate rate ratio for severe disease was 3.21 (95% CI 3.01 to 3.41, *p*<0.001) in those with moderate risk conditions and 6.3 (95% CI 5.8 to 6.8, *p*<0.001) in those eligible for shielding. The rate ratio associated with eligibility for shielding was 6.1 (95% CI 5.4 to 7.0) up to the end of August 2020 and 6.4 (95% CI 5.8 to 7.0) after August 2020.

**Table 2:** Rate ratios for severe COVID-19 in those not resident in care homes

			Univariate		Multivariable	
	Controls (86,902)	Cases (7217)	Rate ratio (95% CI)	*p*-value	Rate ratio (95% CI)	*p*-value
No risk condition	45,354 (52%)	1897 (26%)		.		.
Moderate risk condition	33,909 (39%)	3702 (51%)	3.20 (3.01, 3.41)	<0.001	2.67 (2.50, 2.85)	<0.001
Shielding eligibility category						
Solid organ transplant	112 (0%)	59 (1%)	13.4 (9.6, 18.8)	<0.001	7.0 (4.6, 10.4)	<0.001
Specific cancers	907 (1%)	240 (3%)	7.9 (6.8, 9.3)	<0.001	3.47 (2.87, 4.19)	<0.001
Severe respiratory	3751 (4%)	733 (10%)	5.8 (5.2, 6.4)	<0.001	4.32 (3.87, 4.83)	<0.001
Rare diseases	341 (0%)	69 (1%)	7.0 (5.3, 9.3)	<0.001	4.68 (3.37, 6.49)	<0.001
On immunosuppressants	742 (1%)	126 (2%)	4.61 (3.76, 5.65)	<0.001	3.26 (2.59, 4.10)	<0.001
Additional conditions	1786 (2%)	391 (5%)	6.6 (5.8, 7.5)	<0.001	4.31 (3.72, 5.00)	<0.001
At least one child under 5	1759 (2%)	150 (2%)	0.87 (0.73, 1.04)	0.1	0.73 (0.60, 0.89)	0.002
Number of school age children in household						
0 school age	80,885 (93%)	6685 (93%)		.		.
1 school age	1749 (2%)	93 (1%)	0.54 (0.43, 0.67)	<0.001	0.76 (0.60, 0.96)	0.02
2 or more	4268 (5%)	439 (6%)	1.06 (0.95, 1.19)	0.3	0.71 (0.62, 0.80)	<0.001
Number of adults in household						
1 adult	62,011 (72%)	4259 (59%)		.		.
2 adults	19,190 (22%)	1951 (27%)	1.57 (1.48, 1.67)	<0.001	1.80 (1.68, 1.92)	<0.001
3 to 4	4611 (5%)	769 (11%)	2.50 (2.28, 2.74)	<0.001	3.19 (2.87, 3.54)	<0.001
5 to 9	326 (0%)	85 (1%)	3.98 (3.10, 5.11)	<0.001	5.8 (4.3, 7.7)	<0.001
10 or more	247 (0%)	102 (1%)	10.6 (7.6, 14.6)	<0.001	11.4 (8.0, 16.2)	<0.001
SIMD quintile						
1 - most deprived	21,388 (25%)	2162 (30%)		.		.
2	19,072 (22%)	1761 (24%)	0.91 (0.85, 0.98)	0.01	0.95 (0.88, 1.03)	0.3
3	15,399 (18%)	1264 (18%)	0.76 (0.70, 0.82)	<0.001	0.84 (0.76, 0.92)	<0.001
4	14,870 (17%)	1079 (15%)	0.65 (0.59, 0.70)	<0.001	0.72 (0.65, 0.79)	<0.001
5 - least deprived	16,084 (19%)	944 (13%)	0.53 (0.48, 0.58)	<0.001	0.60 (0.53, 0.66)	<0.001
Occupation						
Other / undetermined	84,672 (98%)	6962 (97%)		.		.
Teacher	336 (0%)	11 (0%)	0.35 (0.19, 0.64)	<0.001	0.42 (0.22, 0.81)	0.01
Health care, not PF / undetermined	826 (1%)	87 (1%)	1.18 (0.94, 1.47)	0.2	1.20 (0.94, 1.53)	0.2
Health care PF	846 (1%)	145 (2%)	1.80 (1.50, 2.16)	<0.001	1.86 (1.52, 2.27)	<0.001
Recent hospital visit/stay	3565 (4%)	2415 (33%)	12.3 (11.5, 13.2)	<0.001	10.2 (9.5, 11.0)	<0.001

Severe COVID-19 is defined by entry to critical care or fatal outcome

Percentages are column percentages for each variable

*PF*, patient-facing

Rate ratios are from conditional logistic regression models with cases and controls matched for age, sex and general care practice

Univariate rate ratios are for models with a single covariate

Multivariable rate ratios are for a model including all covariates shown in the table

Among those eligible for shielding, solid organ transplant recipients were the group at highest risk, with a univariate rate ratio of 13.4 (95% CI 9.6 to 18.8, *p*<0.001) for severe COVID-19. On multivariable adjustment for the covariates shown in Table 2, the rate ratios for all these shielding groups remained high.

We examined the time course of the rate ratio associated with eligibility for shielding. As shown in Additional file 1: Table S2, most of the solid organ transplant recipients, those with severe respiratory disease and cancer patients were included in the first batch of shielding letters sent on 3 April 2020. Additional file 1: Figure S1(a) shows that the rate ratio associated with eligibility for shielding increased from 5.17 in the time window with mid-point 1 April to 8.89 in the time window with mid-point 1 May. The rate ratio associated with moderate risk conditions also rose in the first half of May, but fell more rapidly than the rate ratio associated with eligibility for shielding. Thus, there was no evidence that shielding advice reduced the rate ratio.

### Associations of severe COVID-19 with transmission-related factors

#### Associations in those eligible for shielding

Table 3 shows associations with risk factors among those eligible for shielding only, with severe respiratory disease (the largest category) as reference category. The rate ratio increased with the number of adults in the household but not with the number of children. The strongest risk factor for severe COVID-19 among those eligible for shielding was recent exposure to hospital, with a rate ratio of 6.0 (95% CI 4.7 to 7.7, *p*<0.001) in the multivariable model which all the covariates shown in the table were entered simultaneously. Of severe cases among those eligible for shielding, 739 (45%) had recent exposure to hospital. Using Miettinen’s formula as given above, the population attributable risk fraction for recent exposure to hospital in those eligible for shielding can thus be calculated as 37% of severe cases.

**Table 3:** Rate ratios for severe COVID-19 associated with risk conditions (with severe respiratory disease as reference category) in those eligible for shielding and not resident in care homes

			Univariate		Multivariable	
	Controls (7808)	Cases (1640)	Rate ratio (95% CI)	*p*-value	Rate ratio (95% CI)	*p*-value
Shielding eligibility category						
Severe respiratory	3834 (49%)	744 (45%)		.		.
Solid organ transplant	112 (1%)	60 (4%)	1.58 (0.90, 2.77)	0.1	1.36 (0.70, 2.63)	0.4
Specific cancers	925 (12%)	240 (15%)	1.28 (0.98, 1.66)	0.07	0.88 (0.64, 1.21)	0.4
Rare diseases	348 (4%)	72 (4%)	1.18 (0.75, 1.84)	0.5	1.14 (0.68, 1.91)	0.6
On immunosuppressants	759 (10%)	129 (8%)	0.74 (0.53, 1.03)	0.07	0.82 (0.56, 1.20)	0.3
Additional conditions	1830 (23%)	395 (24%)	1.19 (0.95, 1.49)	0.1	1.00 (0.77, 1.31)	1
At least one child under 5	66 (1%)	27 (2%)	0.80 (0.32, 2.02)	0.6	0.45 (0.15, 1.31)	0.1
School-age children in household						
0 school age	7537 (97%)	1545 (94%)		.		.
1 school age	82 (1%)	19 (1%)	0.36 (0.13, 0.99)	0.05	0.84 (0.29, 2.45)	0.7
2 or more	189 (2%)	76 (5%)	1.03 (0.60, 1.76)	0.9	0.74 (0.40, 1.37)	0.3
Adults in household						
1 adult	5811 (75%)	1010 (62%)		.		.
2 adults	1647 (21%)	458 (28%)	1.60 (1.30, 1.97)	<0.001	1.74 (1.37, 2.21)	<0.001
3 or more	339 (4%)	167 (10%)	2.15 (1.51, 3.07)	<0.001	2.52 (1.66, 3.82)	<0.001
SIMD quintile (integer)	2 (1–4)	2 (1–4)	0.97 (0.91, 1.05)	0.5	0.98 (0.90, 1.06)	0.6
Recent hospital visit/stay	840 (11%)	739 (45%)	5.8 (4.6, 7.4)	<0.001	6.0 (4.7, 7.7)	<0.001

Severe COVID-19 is defined by entry to critical care or fatal outcome

Percentages are column percentages for each variable

Rate ratios are from conditional logistic regression models matched for age, sex and general practice

Univariate rate ratios are for models with a single covariate

Multivariable rate ratios are for a model including all covariates shown in the table

#### Associations in the overall population

Table 2 shows the association of severe COVID-19 with risk factors in the general population, including those eligible for shielding but excluding residents in care homes. The risk of severe COVID-19 increased with the number of adults in the household, but was inversely associated with the number of school-age children in the household in a multivariable model in which all the covariates in Table 2 were entered simultaneously. The rate ratio associated with two or more adults (with single-adult households as reference category) was 2.08 (95% CI 1.95 to 2.21, *p*<0.001) and the rate ratio associated with one or more school-age children was 0.72 (95% CI 0.64 to 0.81, *p*<0.001). The other demographic factor associated with increased risk was socioeconomic deprivation: the rate ratio in the least deprived quintile compared with the most deprived quintile was 0.60 (95% CI 0.53 to 0.66, *p*<0.001). As shown in Additional file 1: Figure S1(b), in a joint model with number of adults in household, number of children in household, and SIMD deprivation score the rate ratio for severe disease per adult in household remained in the range 1.5 to 2 throughout the epidemic, and the rate ratio per school-age child in household remained mostly in the range 0.7 to 1.

As reported previously, in comparison with other occupations patient-facing health-care workers were at higher risk of severe disease with a rate ratio of 1.80 (95% CI 1.50 to 2.16, *p*<0.001), and teachers were at lower risk with a rate ratio of 0.35 (95% CI 0.19 to 0.64, *p*<0.001).

##### Association with recent exposure to hospital

As Table 2 shows, the strongest risk factor (in terms of deviance explained) for severe COVID-19 in the overall population was recent exposure to hospital: rate ratio 12.3 (95% CI 11.5 to 13.2, *p*<0.001). Exclusion of the 11% of those dying within 28 days of a positive test who did not have COVID-19 as underlying cause of death on their death certificates changed this rate ratio only slightly to 11.9 (11.0, 12.8).

**Fig. 2:**
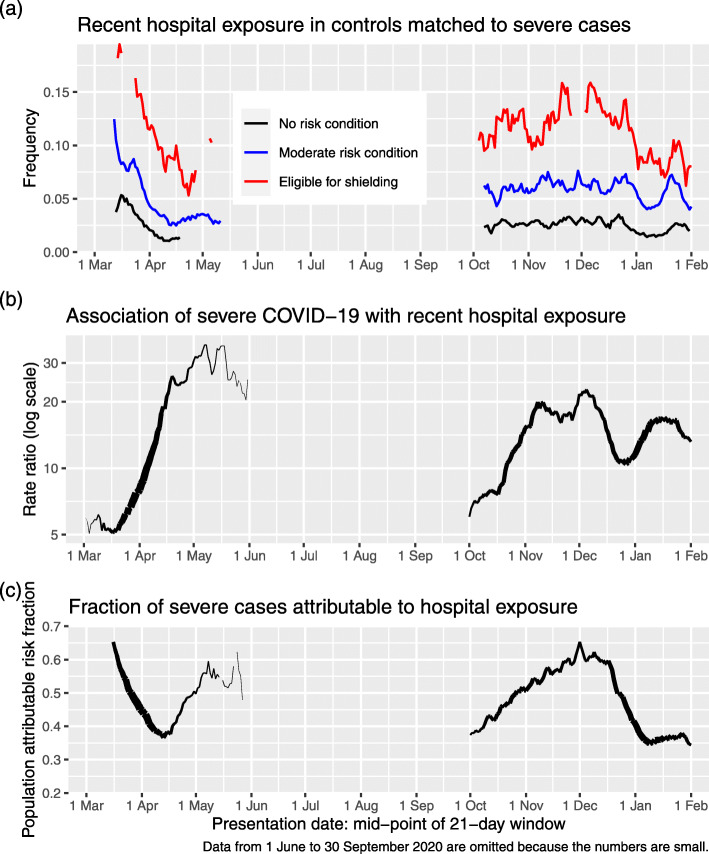
**a** Recent hospital exposure by risk group and 7-day sliding window of presentation dates. **b** Rate ratio for severe COVID-19 associated with recent hospital exposure in those not resident in care homes. **c** Fraction of severe cases attributable to recent hospital exposure in those not resident in care homes

Figure 2a shows the time course of the frequency of recent exposure to hospital using data from controls; for monitoring background exposure levels, it is appropriate to examine the frequency in controls rather than in cases. Recent exposure to hospital fell precipitously when restrictions on non-COVID-19 admission were imposed in March, but remained higher in those eligible for shielding than in those with moderate risk conditions or no risk conditions throughout.

As shown in Fig. 2b, the rate ratio associated with recent exposure to hospital increased from 8 in the time window with mid-point 1 April to a rate ratio of 31 in the time window with mid-point 1 May.

The population attributable risk fraction of severe COVID-19 for recent exposure to hospital in the general population can be calculated as 30% over the study period. This fraction reached a peak in the first wave of 50% of severe cases at the beginning of May, declined rapidly over the next few weeks and increased again to 65% of severe cases at the beginning of December (Fig. 2c).

In a *post hoc* analysis, we found that the association of recent exposure to hospital with severe disease was largely driven by inpatient exposure [rate ratio 33.6 (95% CI 30.4 to 37.3, *p*<0.001)]; the rate ratios associated with day case exposure [2.59 (95% CI 1.87 to 3.58, *p*<0.001)] or outpatient exposure [2.67 (95% CI 2.40 to 2.97, *p*<0.001)] were much lower. To test if the association of severe COVID-19 with recent inpatient exposure was likely to simply reflect confounding by time invariant comorbidity, the association was examined by time window as described in the “Methods” section. Table 4 shows that the rate ratio for severe COVID-19 associated with inpatient exposure only in the recent interval (days 5 to 14 before testing positive), with exposure only in the less recent interval (days 15 to 24 before testing positive) as reference category was 5.9 (95% CI 3.6 to 9.7, *p*<0.001).

**Table 4:** Rate ratios for severe COVID-19 by time window of hospital in-patient exposure, excluding care home residents

Time window of exposure	Controls	Cases	Rate ratio (95% CI)	*p*-value
Less recent interval only	494 (29%)	147 (6%)		.
Recent interval only	470 (28%)	799 (35%)	5.9 (3.6, 9.7)	<0.001
Both intervals	741 (43%)	1335 (59%)	6.9 (4.3, 11.1)	<0.001

Rate ratios are from conditional logistic regression models

Dataset restricted to those exposed at some time between 5 and 24 days before first testing positive

Recent interval = days 5 to 14 before first testing positive, less recent interval = days 15 to 24 before first testing positive

Reference category is exposure in less recent interval only

The frequency of recent inpatient exposure over the period of study in cases not resident in care homes was 28%. From this and the rate ratio of 33.6, the population attributable risk fraction for recent hospital inpatient exposure can be calculated from Miettinen’s formula as 27%. As Additional file 1: Table S3 shows, most of the difference in inpatient exposure classification of severe cases between our definition and the ECDC definition arises because cases with onset after hospital discharge would be classified as community onset by the ECDC classification: only 57% of severe cases with inpatient exposure 5–14 days before COVID-19 presentation would be defined as probable or definite hospital-acquired infection (in hospital for at least 8 days before first testing positive) by the ECDC classification. The rate ratio for severe disease associated with being in hospital for at least 8 days before presentation date was 45.4.

## Discussion

### Summary of findings and comparison with other studies

Key findings of this study were (1) that the rate ratios for severe COVID-19 associated with shielding conditions remained high through the epidemic; (2) that recent hospital exposure and the number of adults in the household were associated with severe COVID-19 among those eligible for shielding and also in the general population; and (3) that among those not resident in care homes the proportion of severe cases attributable to hospital attendance reached a peak of 65% during the second wave.

#### Risk to those eligible for shielding

In comparison with those with no risk condition, the highest rate ratio for severe COVID-19 was the 13-fold rate ratio in solid organ transplant recipients. For other conditions deemed eligible for shielding, the rate ratios were between 5 and 8, compared to the rate ratio of about 3 associated with conditions designated as moderate risk, which include heart disease, diabetes, chronic kidney disease and disabling neurological conditions. The numbers of pregnant women with heart disease were too small for the risk in this group to be estimated. Other studies of outcome of COVID-19 in solid organ transplant recipients and other individuals using immunosuppressants have been based only on patients admitted to hospital [12, 13]. Such studies cannot assess the risks to immunosuppressed individuals in the population.

The shielding programme was introduced at a time when general social distancing measures were being introduced and after the first lockdown had started. We had hypothesised a priori that a fall in the rate ratio for severe disease associated with eligibility for shielding within 2 weeks of the first letters being sent out would be consistent with the shielding programme having some impact beyond the population-wide lockdown that had been imposed by this time. However, we did not find this; while presentations of severe cases fell rapidly in the general population from the beginning of April 2020, the fall in presentations among those eligible for shielding advice was delayed so that the rate ratio for severe COVID-19 associated with eligibility for shielding rose during April 2020. Daily deaths fell during April 2020 both in those eligible and those ineligible for shielding. As a lockdown on the general population had been imposed on 23 March 2020, and individuals who considered themselves to be at high risk would have been likely to reduce their contact level before then, there were limited possibilities for risk in this group to be reduced further by advice to shield and offers of help in letters sent from 3 April onwards.

Although we did not find any evidence that the shielding programme *per se* reduced COVID-19 rates, it is possible that without shielding advice and support the outcome in this group would have been worse. It is still relevant to examine why advice to shield, combined with offers of support for delivery of food and medicines, failed to protect some individuals who were identified as clinically extremely vulnerable. The association of severe COVID-19 with recent hospital exposure in those who were eligible for shielding, together with the increase in the rate ratio associated with this exposure during periods when population-wide social distancing measures were being imposed, suggests that exposure to transmission in hospital settings is at least part of the explanation. Our finding that the risk of severe COVID-19 was increased in those who were sharing a household with other adults suggests that this was another constraint on the effectiveness of advice to shield, as no support was provided for other household members to co-isolate with the vulnerable individual. A survey conducted online in the first 2 weeks of June 2020 found that 65% of those living alone or sharing a household with one other person reported that they followed the guidance completely, but only 55% of those living with two or more others did [14]. Development of policies to overcome these constraints may lay a basis for focused protection of the most vulnerable individuals to be more effective in future epidemics.

#### Association with occupation and with household composition

The increased risk of severe COVID-19 in patient facing health care workers is consistent with our previous report of a threefold risk for hospitalised COVID-19 earlier in the epidemic [6]. We and others have previously reported lower risk in teachers compared with others of the same age and sex [7, 15]. The inverse association of severe disease with the number of school-age children in the household extends and confirms the findings of an earlier study of health care workers and their families [16]. In the OPENSAFELY cohort, the rate ratio for fatal COVID-19 associated with living with children aged 0–11 years was 0.75 after adjusting for covariates, but no dose-response relationship was reported [17].

The strong association of severe disease with number of adults in the household is consistent with a recent estimate, based on an intensive study of households in the Netherlands, that the secondary attack rate for SARS-CoV-2 infection is as high as 51% in adults [18]. We would expect the risk of infection to increase with the number of other people in the household, especially where population-wide social distancing between households has been imposed. We might also expect that this association would be strongest for severe disease, as the infecting dose is likely to be higher in intra-household transmissions than in transmissions between households. This is consistent with classic studies of other viral infections showing that secondary cases in a household are more severe than index cases [19]. The inverse association of severe COVID-19 with number of children in the household, and the lower risk in teachers however suggests that current or past exposure to children may have some protective effect on adults. A possible explanation may be that other coronaviruses generate cross-reactive T-cell responses that may confer some resistance to SARS-CoV-2 [20]. From a public health perspective the most relevant implication is that although the rate ratio per child in the household has been higher in the second wave of the epidemic than in the first wave, it has remained below 1 in almost all time windows.

#### Association with recent exposure to hospital

A striking finding from our analysis is the population attributable risk fraction of 30% for severe disease associated with recent exposure to hospital. Although we had pre-specified this category to include day case and outpatient exposure, the association was driven by inpatient exposure. The population attributable risk fraction associated with inpatient exposure was 27%. The calculation of the population attributable risk fraction for an exposure provides an upper bound on the predicted effect of removing that exposure. As explained in the “Methods” section, for estimating the population attributable risk fraction, it is appropriate to use an inclusive definition of exposure that includes all those whose infection could have been acquired in hospital. Using the ECDC definition of probable/definite nosocomial acquisition would give a lower estimate of the proportion of severe cases exposed to hospitals and the population attributable risk fraction, because the ECDC definition excludes cases who were not continuously in hospital for at least 8 days before presenting.

The association of severe COVID-19 with recent hospital admission is likely to be confounded by pre-existing risk conditions. However adjusting for risk conditions in a multivariable analysis reduced the rate ratio associated with recent hospital exposure only slightly (from 12.3 to 10.2). The most compelling evidence of causality is the time window analysis, which shows that the association of disease is with elapsed time since exposure corresponding to the known incubation period of SARS-CoV-2. The rate ratio estimated from the time window analysis is not directly comparable with the rate ratio based on the case-control analysis because restriction to those with discordant exposure between time windows selects those with fewer days in hospital (lower dosage of the exposure).

The role of nosocomial transmission was recognised early in the COVID-19 epidemic. Hospital-acquired infection was suspected in 41% of hospitalised cases seen in January 2020 in one centre in Wuhan [21]. A commentary in April 2020 noted that “in Lombardy, SARS-CoV-2 became largely a nosocomial infection” [22]. A study of 11 hospitals (10 in the UK) reported that 12.5% of hospitalised cases of COVID-19 up to the end of April 2020 met the ECDC criterion for definite nosocomial infection [23]. Most other reports have used the ECDC criterion for probable/definite nosocomial infection. A study presented to the UK Scientific Advisory Group on Emergencies in January 2021 based on national data for hospital episodes and COVID-19 tests in England linked to symptom onset data from the COVID-19 Clinical Information Network estimated that 27.6% of hospitalised cases up to the end of July 2020 met this criterion and that onward transmission of COVID-19 from hospital-acquired cases could account for another 5% of hospitalised cases [24]. No systematic study of nosocomial cases has been reported for England in the second wave: from routinely reported data the proportion of hospitalised cases that met the criterion for probable/definite nosocomial was estimated to be 18% in October 2020. For Scotland, the ARHAI (Antimicrobial Resistance and Healthcare Associated Infection) unit of NHS National Services Scotland recently reported that among those who died within 28 days of testing positive for COVID-19 (including care home residents) during 2020, 30% met the ECDC definition of probable or definite nosocomial infection [25].

### Strengths and limitations

Strengths of our study are the national coverage and the comprehensive linkage to medical records and demographic risk factors. A limitation is that we do not have primary care data other than encashed prescriptions. Furthermore, as most immunosuppressant drugs are prescribed through hospitals where linkage to prescribing records is not yet possible, the risks associated with specific immunosuppressant drug classes could not be investigated. We have no data on help with daily activities from non-resident carers as another possible source of exposure of clinically vulnerable individuals attempting to shield themselves. As we do not have individual-level data on compliance with shielding advice, we cannot directly evaluate the extent to which non-compliance or non-receipt of letters might have reduced the effectiveness of shielding advice: we can only evaluate the programme as implemented.

As all hospital inpatients are tested for SARS-CoV-2 every few days and all deaths within 28 days of a positive test are officially classified as deaths involving COVID-19, some misclassification of deaths from other causes as COVID-19 deaths is likely in those with recent hospital exposure. However, the rate ratio associated with recent hospital exposure was barely changed when fatal cases without COVID-19 as underlying cause of death on death certificate were omitted.

### Policy implications

Our results have implications for policies on shielding the vulnerable, vaccination and control of SARS-CoV-2 transmission in the population. We have identified two sources of exposure that are associated with severe disease and cannot easily be avoided by those advised to shield: in-patient hospital care and sharing a household with other adults. Measures to allow vulnerable individuals to reduce their exposure could include extra protection from nosocomial infection and support for other household members to co-isolate with the vulnerable individual.

Our findings support the policy of assigning highest priority for vaccination to those with risk conditions eligible for shielding, as these groups have markedly elevated risks of severe COVID-19. For solid organ transplant recipients—the group at highest risk—it is uncertain whether the vaccine will evoke an immune response sufficient to be effective [26]. As there is now evidence that vaccination reduces transmission to household contacts [27], one way to reduce risk in this group would be to vaccinate their household contacts.

Policies for control of transmission in the population and for reducing burden on health services and total deaths have focused on population-wide reduction of social contact. However our analysis provides compelling evidence for a substantial contribution of nosocomial transmission to the burden of severe COVID-19 even during the second wave. A report for NHS England by the Healthcare Safety Investigation Branch noted the challenges of controlling nosocomial transmission and recommended that a national strategy for infection prevention and control should be developed [28]. Vaccination of health care workers is likely to reduce nosocomial transmission, but vaccination of those booked for elective procedures should also be considered. We have not attempted to quantify the onward transmission of COVID-19 from discharged patients, but the PHE modelling study estimated that this could have accounted for 5% of cases requiring hospitalisation in England during the first wave [24]. This suggests more stringent testing before discharge and quarantine post discharge should be considered. More detailed understanding of how recommended infection control policies are being operationalised is also needed [29].

## Conclusions

The effectiveness of shielding vulnerable individuals was limited by the inability to control transmission in hospital and from other adults in the household. Mitigating the impact of the epidemic requires control of nosocomial transmission.

## Supplementary Information

**Additional file 1** Supplementary Figure and Tables.

## Data Availability

The component datasets used here are available via the Public Benefits and Privacy Panel for Health and Social Care https://www.informationgovernance.scot.nhs.uk/pbpphsc/ for researchers who meet the criteria for access to confidential data. All source code used for derivation of variables, statistical analysis and generation of this manuscript is available on https://github.com/pmckeigue/covid-scotland_public.

## Abbreviations

CHI: Community Health Index
ECDC: European Centre for Disease Prevention and control
ECOSS: Electronic Communication of Surveillance in Scotland
NHS: National Health Service
NRS: National Records of Scotland
PHS: Public Health Scotland
SICSAG: Scottish Intensive Care Society and Audit Group
SIMD: Scottish Index of Multiple Deprivation
SMR: Scottish Morbidity Record
