# Supplementary material for: Relation of severe COVID-19 in Scotland to transmission-related factors and risk conditions eligible for shielding support: REACT-SCOT case-control study
Source: BMC Med. 2021 Jun 23;19:149. doi: 10.1186/s12916-021-02021-5 (PMC8219469; doi:10.1186/s12916-021-02021-5)
Supplement: Supplementary file 1 — Additional file 1 Supplementary Figure and Tables. [file 12916_2021_2021_MOESM1_ESM.pdf]

Supplementary Material

Supplementary Figures

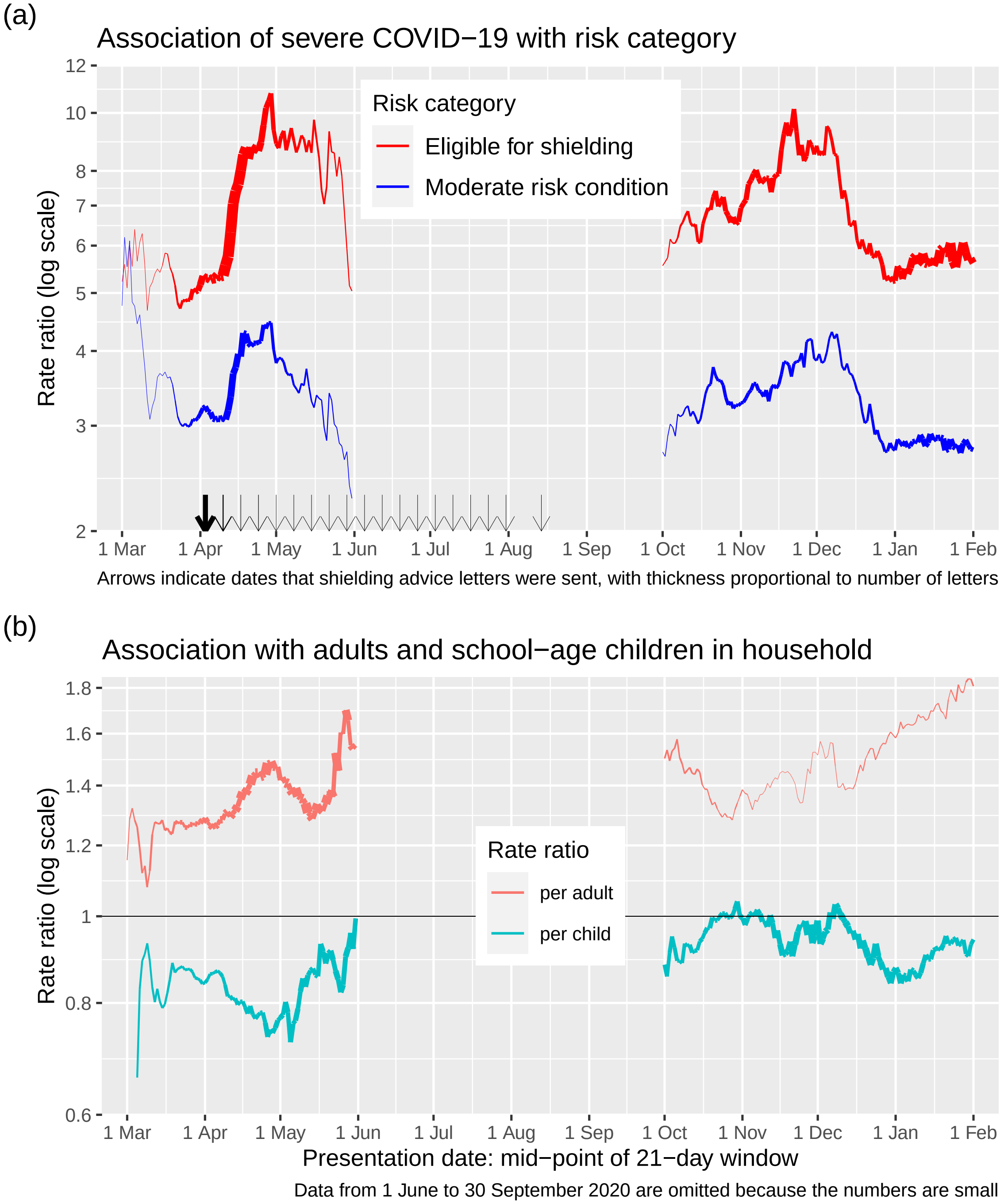

Fig S1. (a) Rate ratios for severe COVID-19 excluding care home residents, by risk group and sliding window of presentation dates. (b) Rate ratio for severe COVID-19 per adult and per child in household, excluding care home residents, in model with number of adults, number of children and SIMD deprivation score as covariates. Line thickness is proportional to precision of estimate. Labels on the y axes are untransformed rate ratios, though the axis scales are log-transformed

Supplementary Tables

Table S1. Shielding eligibility cohort by eligibility category and age

	Age group				
	0-39	40-59	60-74	75 or more	All
**Shielding eligibility category**					
Solid organ transplant	1170 (17%)	2644 (38%)	2520 (37%)	537 (8%)	6872
Specific cancers	1417 (5%)	6609 (25%)	11303 (42%)	7367 (28%)	26699
Severe respiratory	3646 (4%)	18465 (21%)	37221 (43%)	27261 (31%)	86599
Rare diseases	2527 (23%)	3092 (28%)	2557 (23%)	2894 (26%)	11072
On immunosuppressants	6465 (20%)	10754 (34%)	9577 (30%)	5010 (16%)	31807
Additional conditions	6087 (12%)	13870 (28%)	15791 (32%)	13842 (28%)	49595
All shielding categories	21312 (10%)	55434 (26%)	78969 (37%)	56911 (27%)	212644

Percentages are row percentages

Table S2. Severe test-positive cases classified by hospital onset status (as defined by ECDC) and recent inpatient exposure

	No recent hospital exposure	Recent hospital exposure
Community onset	6464 (94%)	569 (27%)
Non-hospital onset	279 (4%)	66 (3%)
Indeterminate hospital onset	107 (2%)	270 (13%)
Probable hospital onset	0 (0%)	434 (20%)
Definite hospital onset	0 (0%)	794 (37%)
All ECDPC categories	6850	2133

Percentages are column percentages

ECDC, European Centre for Disease Prevention and Control

Table S3. Dates of sending advice letters to those eligible for shielding

	3 Apr	10 Apr	17 Apr	24 Apr	1 May or later	All
Solid organ transplant	6674 (97%)	10 (0%)	8 (0%)	22 (0%)	143 (2%)	6857
Specific cancers	17485 (78%)	241 (1%)	556 (2%)	897 (4%)	3132 (14%)	22311
Severe respiratory	77389 (90%)	424 (0%)	705 (1%)	2170 (3%)	4837 (6%)	85525
Rare diseases	916 (11%)	6363 (76%)	159 (2%)	474 (6%)	444 (5%)	8356
On immunosuppressants	13302 (43%)	9806 (32%)	4007 (13%)	1658 (5%)	2067 (7%)	30840
Additional conditions	2287 (5%)	7290 (17%)	6626 (16%)	7554 (18%)	18024 (43%)	41781
All shielding categories	118053 (60%)	24134 (12%)	12061 (6%)	12775 (7%)	28647 (15%)	195670

Percentages are row percentages
